# Nitrogen removal capability and mechanism of a novel low-temperature-tolerant simultaneous nitrification-denitrification bacterium *Acinetobacter kyonggiensis* AKD4

**DOI:** 10.3389/fmicb.2024.1349152

**Published:** 2024-09-10

**Authors:** Jiwei Mao, Ruojin Zhao, Yiyi Li, Wenpan Qin, Shengchun Wu, Weiping Xu, Peng Jin, Zhanwang Zheng

**Affiliations:** ^1^School of Environmental & Resource, Zhejiang A & F University, Hangzhou, China; ^2^Zhejiang Sunda Public Environmental Protection Co., Ltd., Hangzhou, China; ^3^College of Food and Health, Zhejiang A & F University, Hangzhou, China

**Keywords:** simultaneous nitrification and denitrification, low temperature, transcriptomics, cell growth, nitrogen metabolism

## Abstract

A low-temperature-tolerant simultaneous nitrification-denitrification bacterial strain of *Acinetobacter kyonggiensis* (AKD4) was identified. It showed high efficiency in total nitrogen (TN) removal (92.45% at 10°C and 87.51% at 30°C), indicating its excellent low-temperature tolerance. Transcriptomic analysis revealed possible metabolic mechanisms under low-temperature stress. Genes involved in cell growth, including ATP synthase (*atpADGH*), amino acid (*glyA*, *dctA*, and *ilvE*), and TCA cycle metabolism (*gltA*, *fumC*, and *mdh*) were remarkably upregulated from 1.05–3.44-fold at 10°C, suggesting that their actions enhance survivability at low temperatures. The expression levels of genes associated with nitrogen assimilation (*glnAE*, *gltBD*, and *gdhA*), nitrogen metabolism regulation (*ntrC*, *glnB*, and *glnD*), and denitrification processes (*napA*) were increased from 1.01–4.38-fold at 10°C, which might have contributed to the bacterium’s highly efficient nitrogen removal performance at low temperatures. Overall, this study offers valuable insights into transcriptome, and enhances the comprehension of the low-temperature-tolerant mechanism of simultaneous nitrification and denitrification processes.

## Graphical abstract

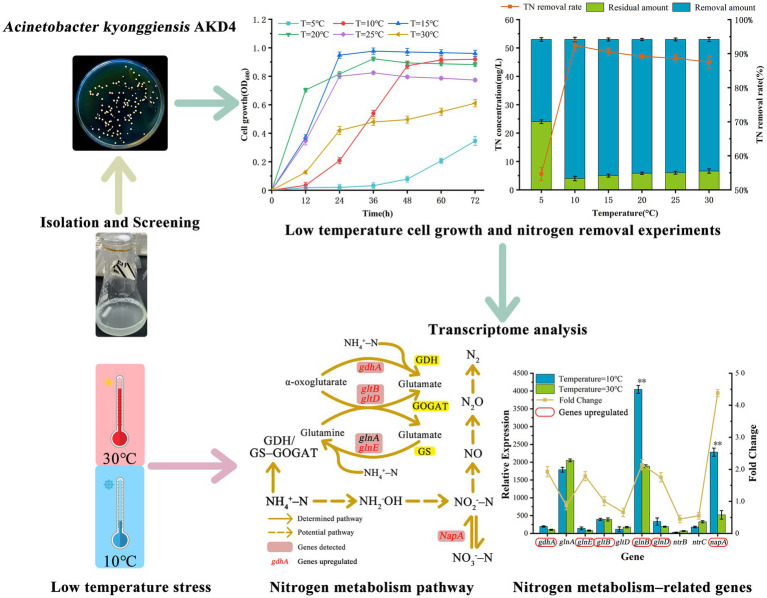

## 1 Introduction

In recent years, environmental problems caused by the excessive emission of nitrogen compounds in nature have intensified. For example, direct pollution of the water environment, leading to eutrophication of water bodies, poisoning and threatening the survival of aquatic organisms, disrupting the ecological balance and further jeopardizing human health (Basu et al., 2022). The control and purification of nitrogen in wastewater also receive continuous attention. Compared with the methods of physical and chemical denitrification, biological denitrification method has been widely used in wastewater denitrification treatment with the characteristics of cost-effective, safe and stable operation, and no secondary pollution (Zhang et al., 2023). However, traditional biological denitrification facilities cover a large area and the operation process is complicated, resulting in an increase in construction and operation costs (Hao et al., 2022; Yan et al., 2022). Therefore, exploring a more cost-effective biological denitrification technology has scientific value and practical significance.

It was found that heterotrophic nitrifying bacteria, *Arthrobacter* sp., showed faster growth rate, better environmental adaptability, and tolerance to high dissolved oxygen environments, compared with autotrophic nitrifying bacteria commonly used in the traditional biological nitrogen removal process in the past (Li et al., 2015). In 1983, Robertson successfully isolated the denitrifying bacterium *Thiosphaera pantotropha* from activated sludge, as this strain could regulate its own metabolic activity and neutralize the acidic and alkaline substances that were produced in the process of nitrification and denitrification, it made it possible to achieve simultaneous nitrification and denitrification (SND) in a single reactor, which largely saved the construction cost (Yan et al., 2022). Since then, the SND capable strains have begun to attention, the global scholars have discovered and isolated a large number of SND capable strains, such as *Acinetobacter*, *Bacillus*, *Cupriavidus*, *Halomonas*, *Klebsiella*, *Marinobacter*, *Pseudomonas*, and *Photobacterium* (Huang et al., 2020). It was found that SND bacteria could maintain high activity under high salinity (Duan et al., 2015), nutrient-poor (Guo et al., 2013), high NH_4_^+^–N (Shoda and Ishikawa, 2014), and extreme pH (Wang et al., 2016). For example, *Pseudomonas* sp. DM02 was able to completely remove the 10 mg/L of NH_4_^+^–N after 12 h of incubation under low nutrient supply conditions, showing a good performance in nutrient-poor growth and efficient nitrogen removal (Deng et al., 2021). In a study conducted by Shoda and Ishikawa (2014) on *Alcaligenes* No. 4, which is tolerant to high NH_4_^+^–N environments, it was found that the strain was able to remove more than 90% of NH_4_^+^–N after 24 h of incubation under extreme conditions with an initial NH_4_^+^–N concentration of 900 mg/L.

Up to date, most of the SND strains that have been reported to have potential for application in wastewater treatment systems are mesophilic bacteria, which typically have an optimal denitrification temperature range between 20–37°C, and the strains were nearly not grown at 10°C (Dong et al., 2023). Although some low-temperature nitrogen-removing bacteria have been reported in recent years, the key genes involved in their physiological activity and nitrogenous metabolism and how they respond to low-temperature stress remain unclear. Thus, understanding the low-temperature tolerance and highly efficient nitrogen-removal mechanism of low-temperature SND bacteria is essential.

In the present study, a novel SND bacterium (AKD4) was isolated and identified from activated sludge. The nitrogen-removal performance of AKD4 at low temperatures was investigated, and the relative expression levels of functional genes at different temperatures were analyzed by using transcriptomics. The possible regulatory mechanisms under low-temperature stress were demonstrated. AKD4 has potential for domestic wastewater treatment at low temperatures and during winter. This work enhances understanding of the low-temperature tolerance mechanism of SND bacteria at gene metabolism levels.

## 2 Materials and methods

### 2.1 Culture medium

The LB medium consisted of (g/L) yeast powder (5 g), tryptone (10 g), and NaCl (10 g). The enrichment medium (EM) consisted of (g/L) yeast powder (1 g), peptone (5 g), and KNO_3_ (1 g). The nitrification medium (NM) consisted of (g/L) sodium succinate (5 g), (NH_4_)_2_SO_4_ (0.235 g), Na_2_HPO_4_ (0.5 g), KH_2_PO_4_ (0.5 g), MgSO_4_·7 H_2_O (0.4 g), and trace salt solution (2 mL). The denitrification medium (DM) consisted of (g/L) sodium succinate (5 g), KNO_3_ (0.36 g), Na_2_HPO_4_ (0.5 g), KH_2_PO_4_ (0.5 g), MgSO_4_·7 H_2_O (0.4 g), and trace salt solution (2 mL). The SND medium (SNDM) consisted of (g/L) sodium succinate (5 g), (NH_4_)_2_SO_4_ (0.235 g), KNO_3_ (0.36 g), Na_2_HPO_4_ (0.5 g), KH_2_PO_4_ (0.5 g), MgSO_4_·7 H_2_O (0.4 g), and trace salt solution (2 mL). The initial pH of all the above media was adjusted to 7.0, and a solid medium with 2% agar was added.

### 2.2 Strain screening and isolation

The activated sludge was obtained from the East Lake of Zhejiang Agricultural and Forestry University, Lin’an District, Hangzhou, Zhejiang Province, China. First, to enrich the bacteria, 10 mL of sludge and 100 mL of liquid EM were added into a 500 mL flask which was shaken at 30°C and 180 rpm for 72 h. Afterwards, domestication and cultivation were performed by inoculating 1 mL of culture into liquid SNDM with shaking at 10°C and 180 rpm for 48 h. This was the first domestication cycle, and domestication and cultivation were performed for five times continuously. The obtained bacterial solution was diluted 5 times with sterilized water, 10-fold each time, and then coated uniformly into NM and DM solid plate media. After 48 h of incubation at 30°C, individual colonies were selected and streaked three times to obtain the purified strain. Several isolated and purified monocolonies were inoculated into 96-well plates of liquid NM, and observed the chromogenic reaction of nitrate and nitrite. Finally, the performance of the strains for SND was evaluated, and a strain with excellent nitrogen removal capability was obtained which was named AKD4 and used as an experimental strain for further study.

### 2.3 Strain identification and phylogenetic tree construction

The isolated and screened SND strains were preliminarily identified. The 16S rDNA gene of the isolated AKD4 was amplified using bacterial 16S rDNA universal primers F27 (5′-AGAGTTTG ATCCTGGCTCAG-3′) and 1492R (5′-GGTTACCTTTACGACTT-3′). The RCR amplification procedure was as follows: 94°C predenaturation for 5 min, 94°C denaturation for 30 s, 55°C annealing for 30 s, and 72°C extension for 2 min for 30 cycles; 72°C extension for 10 min; and 12°C insulation. The 16S rDNA sequence of strain AKD4 was sequenced by Zhejiang Sunya Biotechnology Co., Ltd., China, and has been deposited in the GenBank database under accession number PQ136108. In order to analyze the evolutionary relationship of AKD4 and to determine its taxonomic status, a phylogenetic tree was constructed by neighbor-joining method in MEGA 11 based on known sequences related to this strain in the GenBank database.

### 2.4 Assessment of nitrification and denitrification performance

The seed culture with 1 mL of strain AKD4 was inoculated into 100 mL of SNDM and then incubated at 10°C with 180 rpm shaking to assess its SND ability. Then, AKD4 was incubated at different temperatures to investigate the effect of temperature on the growth and SND performance of AKD4. The above experiments (unless otherwise stated) were carried out under the following conditions: initial ammonia concentration of 50 mg/L, C/N of 8, initial pH of 7.0, incubation temperature of 10°C, and an oscillator speed of 180 rpm. The cell growth was analyzed by using a 600 nm (OD_600_) assay. The concentrations of NH_4_^+^–N, nitrate and nitrite were measured by Nessler’s reagent spectrophotometry at 420 nm, *N*-(1-naphthalene)-diaminoethane spectrophotometry at 410 nm, and UV spectrophotometry at 540 nm. TN was analyzed by a standard UV-spectrophotometric assay (DR6000, Hach, United States). All experiments were performed in triplicate.

### 2.5 Transcriptome analysis

#### 2.5.1 Total RNA extraction

To quantify the message RNA (mRNA) level during nitrogen removal, three parallel samples of strain AKD4 were set up and incubated in LB medium at different temperatures (10°C and 30°C), and bacterial samples were collected at mid-logarithmic growth phase (cultivated 20 h after inoculation). Total mRNA of the strains was extracted using the Bacterial Total RNA Extraction Kit (Zhejiang Sunya Biotechnology Co., Ltd., China) according to manufacturer’s specifications. Total RNA was isolated with the Trizol reagent (Invitrogen Life Technologies, Inc.), after which the concentration, quality and integrity were determined using a NanoDrop spectrophotometer (Thermo Scientific). Three micrograms of RNA were used as input material for the RNA sample preparations.

#### 2.5.2 Transcriptome library preparation and sequencing

Sequencing libraries were generated according to the following steps. Zymo-Seq RiboFree Total RNA Library Kit was used to remove rRNA from total RNA. Random oligonucleotides and SuperScript III were used to synthesize the first strand cDNA then use RNaseH to degrade the RNA strand, and in the DNA polymerase I system, use dNTP with dUTP instead of dTTP as raw material to synthesize the second strand of cDNA. Remaining overhangs were converted into blunt ends via exonuclease/polymerase activities and the enzymes were removed. After adenylation of the 3′ ends of the DNA fragments, Illumina PE adapter oligonucleotides were ligated to prepare for hybridization. To select cDNA fragments of the preferred 400–500 bp in length, the library fragments were purified using the AMPure XP system (Beckman Coulter, Beverly, CA, United States). DNA fragments with ligated adaptor molecules on both ends were selectively enriched using Illumina PCR Primer Cocktail in a 15 cycle PCR reaction. Products were purified (AMPure XP system) and quantified using the Agilent high sensitivity DNA assay on a Bioanalyzer 2100 system (Agilent). The sequencing library was then sequenced on NovaSeq 6000 platform (Illumina).

#### 2.5.3 Data quality control and data mapping analysis

To ensure the accuracy and reliability of the transcriptome sequencing results, the quality information of the raw data in FASTQ format was needed to be calculated. First, the raw data was filtered using fastp (0.22.0) software and the clean data was obtained by removing reads containing adapter, reads containing ploy-N and low-quality reads. The reference genome and gene annotation files were downloaded from the genome website again, and the filtered reads were mapped to the reference genome by the reference genome index built by Bowtie2 (2.5.1).^1^

#### 2.5.4 Expression level and differential expression analysis

The gene reads counted by HTSeq (v0.9.1) were used as the original expression levels of each gene and then analyzed for the expression levels. Expression was normalized using FPKM (fragments per kilobase of exon per million fragments mapped) to make the gene expression levels comparable for different genes and different samples. For differential expression analysis, DESeq (v1.38.3) was conducted to analyze mRNAs with significant changes in expression levels under different temperatures, and transcripts that met the criteria of |log2FoldChange| >1 and *p*-value <0.05 were considered as differentially expressed mRNAs.

#### 2.5.5 GO and KEGG enrichment analysis

GO enrichment analysis of the differential genes was performed using topGO, *p*-values were calculated using the hypergeometric distribution method, standard values of significant enrichment (*p*-value <0.05) were set to screen out GO terms that significantly enriched the differential genes, and to determine the main biological functions of the differential genes. And the Kyoto Encyclopedia of Genes and Genomes (KEGG) pathway enrichment analysis of the differential genes was performed using ClusterProfiler (v4.6.0) software, focusing on the significant enrichment pathway with *p*-value <0.05, which could better elucidate how the differential genes affect the roles of the microbial cellular metabolism, signaling and other complicated biological networks through the regulation of these key pathways.

## 3 Results and discussion

### 3.1 Identification of simultaneous nitrification-denitrification strain

**Figure 1:**
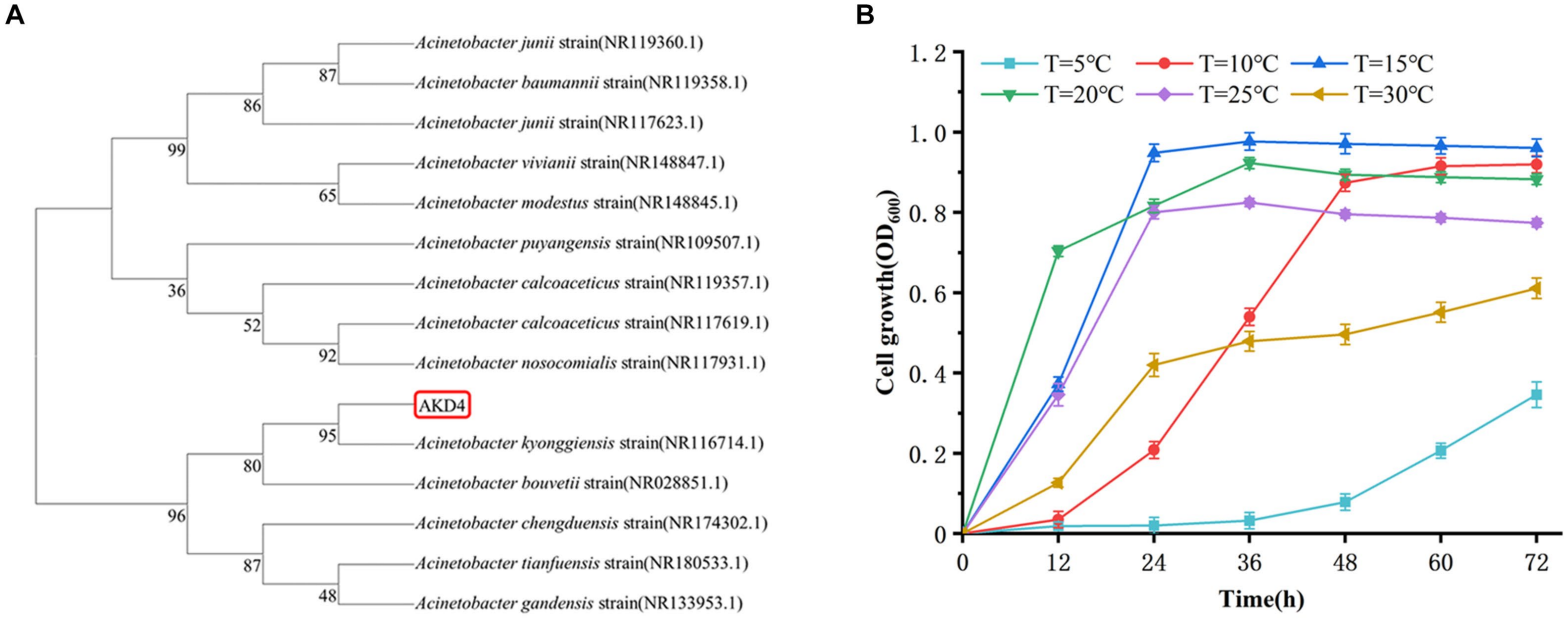
Identification of strain AKD4. **(A)** Phylogenetic tree of strain AKD4. **(B)** Effects of temperature on cell growth.

The strain AKD4, which was screened with high SND performance, was identified by 16S rDNA gene sequence. The sequences obtained were compared and analyzed in the NCBI database using the BLAST tool.^2^ The BLAST database showed that AKD4 had 95% similarity with *Acinetobacter kyonggiensis*. The phylogenetic tree was constructed using MEGA 11 (Figure 1A), which showed that AKD4 and *Acinetobacter kyonggiensis* were in the same branch. Therefore, AKD4 was identified as an *Acinetobacter kyonggiensis* strain.

### 3.2 Effects of temperature on nitrogen removal performance

**Figure 2:**
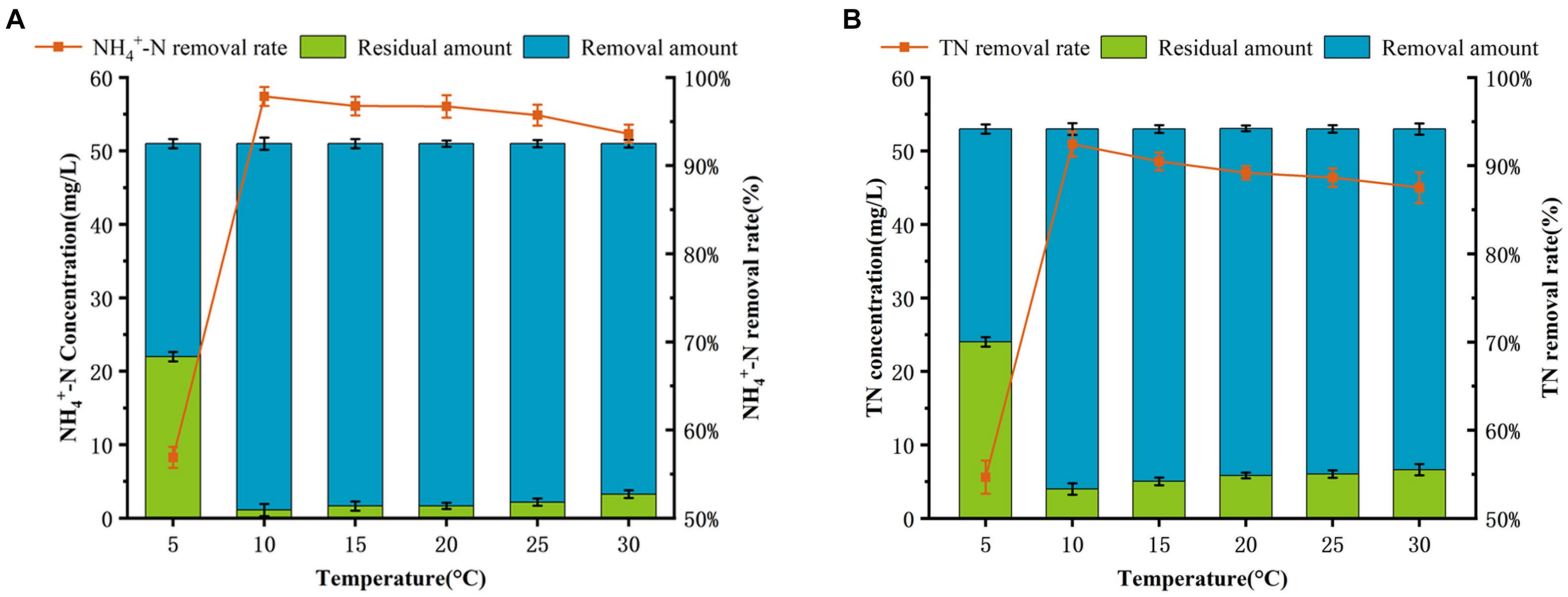
Effects of temperature on the nitrogen removal performance. **(A)** NH_4_^+^–N removal efficiency of AKD4. **(B)** TN removal efficiency of AKD4.

Temperature has significant effect on the performance of nitrogen removal in most SND strains, and their optimal temperatures for cell growth and nitrogen metabolism is 20–37°C (Ji et al., 2015; Wehrfritz et al., 1993). As shown in Figures 1B, 2, AKD4 maintained high cell growth activity and nitrogen removal efficiency at 10°C. The removal efficiency of AKD4 for NH_4_^+^–N and TN reached 97.85 and 92.45%, respectively, after 3 days of incubation (Figure 2A). It was found that the denitrification performance of strain AKD4 was significantly superior to the strain *Pseudomonas tolaasii* Y-11, which was incubated at 15°C for 4 days (He et al., 2016). As shown in Figure 2B, with temperature gradually increased, the TN removal efficiency of AKD4 between 15°C and 25°C remained above 88.65%. Even at 30°C, the TN removal efficiency of AKD4 remained at 87.51%. In addition, although the cell growth of AKD4 decreased slightly when the temperature dropped to 5°C, and the OD_600_ was only 0.346, the removal efficiency of NH_4_^+^–N and TN was still maintained at 56.89 and 54.67%, respectively, after 72 h. Overall, these results indicated that AKD4 is a psychrophilic microorganism and can maintain high activity and nitrogen removal efficiency at low temperatures, and it can be applied to domestic wastewater treatment in winter or under low-temperature conditions.

### 3.3 Determination of gene expression at different temperatures

**Figure 3:**
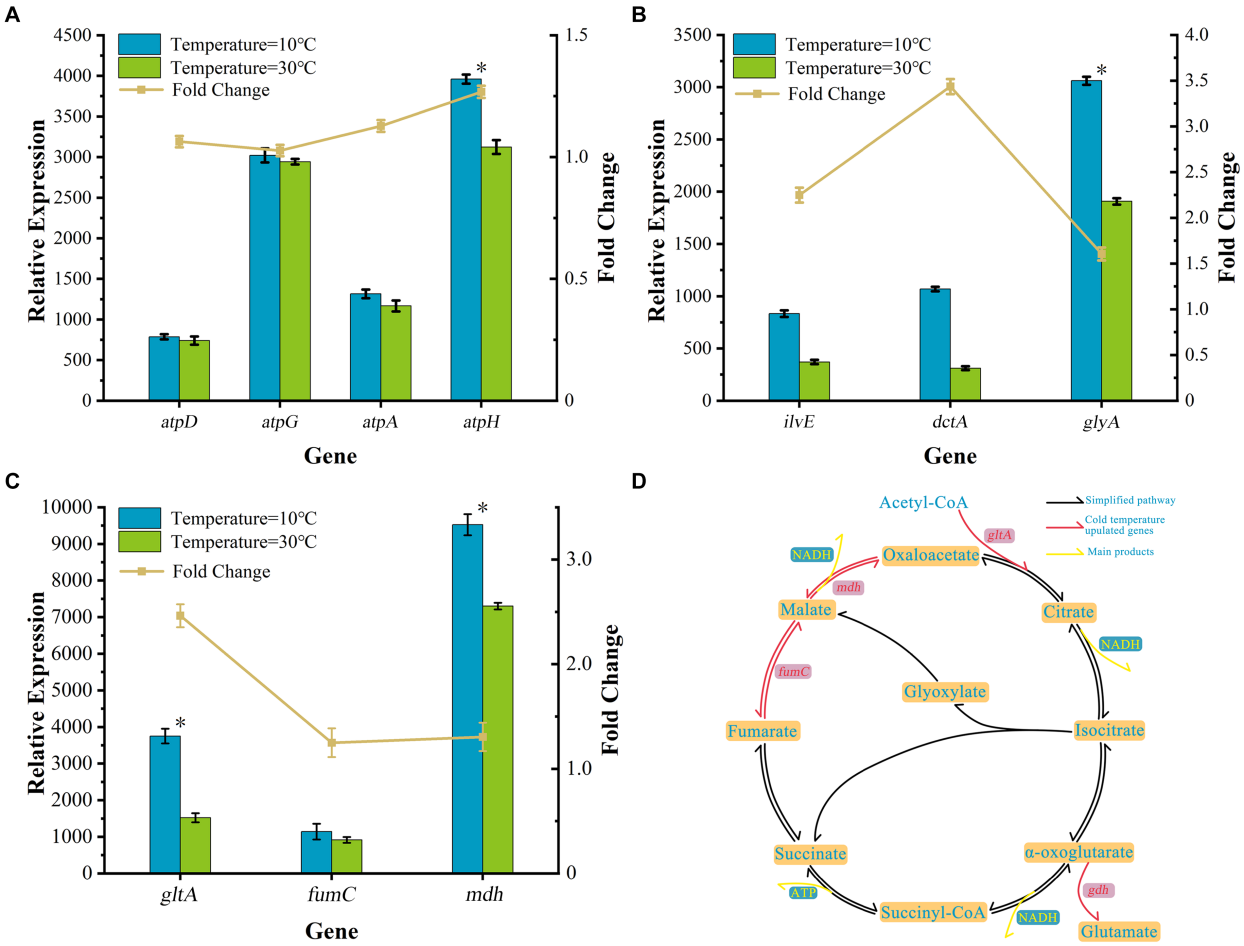
Transcriptional analysis of changes in cell growth gene expression. **(A)** The relative expression levels of four ATP synthase pathway genes. **(B)** The relative expression levels of three amino acid genes. **(C)** Genes involved in the TCA cycle pathway. **(D)** The TCA cycle pathway and glutamate biosynthetic pathway of strain AKD4.

Temperature can directly and strongly affect the activity of AKD4, which seriously affects the nitrogen removal performance. In order to gain a deeper understanding of the temperature effect on the biological nitrogen removal mechanism, the transcriptome analysis of AKD4 was performed at 10°C and 30°C, respectively. Transcriptomic results were annotated and analyzed by using KEGG Automated Annotation Server. As shown in Figure 3, functional genes related to cell growth and nitrogen metabolism in AKD4 showed significant changes at 10°C compared with those at 30°C.

#### 3.3.1 Expression of cell growth-related genes

The low-temperature stress mechanism of cell metabolism was explored. The expression levels of differential genes related to the cell growth and metabolism of AKD4 were analyzed at 10°C and 30°C. At 10°C, the relative expression levels of four ATP synthase genes (*atpA*, *atpD*, *atpG*, and *atpH*) were significantly upregulated 1.13-, 1.06-, 1.03-, and 1.27-fold those at 30°C, respectively (Figure 3A). The ATP synthase plays a crucial role in oxidative phosphorylation, acts as a major intracellular energy donor, provides energy to the cell, and participates in most metabolic reactions in cells. The *atpA* gene is an important catalytic gene for ATP synthase, which has a significant impact on ATP synthase production, and the lack of this gene inhibits the respiration of bacterial strains (Smirnova et al., 2017). The *atpD* gene influences not only ATP synthase production and respiration of bacterial strains but also bacterial biofilm production (Patil and Jain, 2019). Furthermore, *atpG* and *atpH* genes play a vital role in the survival of cells and in catalytic ATP synthase and energy production (Karuppusamy et al., 2018). ATP synthase is closely related to the energy metabolism of organisms and is the main form of energy synthesized by bacteria, enabling them to adapt to cold environments (Christensen and Olsen, 1998; Yang et al., 2019). It cooperates with key genes related to the TCA cycle and amino acid metabolism, which provides microbial cell growth with necessary energy (Bae et al., 2000; Jozefczuk et al., 2010). The upregulation of the relative expression level of ATP synthase in AKD4 at low temperatures may be conducive to promoting the adaptation of strain AKD4 to the low-temperature environment and maintaining an excellent growth metabolism and nitrogen removal performance.

Additionally, the relative expression levels of AKD4 amino acid-related genes *glyA*, *dctA*, and *ilvE* were upregulated 1.60-, 3.44-, and 2.25-fold, respectively (Figure 3B). Amino acids are the main components of proteins and participate in a variety of metabolic pathways to provide intermediate metabolites and precursors for cell growth. The *glyA* gene encodes a serine hydroxy methyltransferase required to catalyze the conversion of serine into glycine, which is a major source of carbon and energy for biological cells. Besides, C4-dicarboxylic acid transporter enzyme, which is encoded by *dctA* and responsible for catalyzing the entry of fumarate into glutamate, is involved in the cell growth of the energy cycle (Youn et al., 2009). Branched chain amino acids (BCAAs) are essential nutrients for cell growth and have several functions, including promoting protein synthesis and regulating glucose metabolism. The BCAAs catalyzed by the branched-chain amino acid aminotransferase encoded by the *ilve* gene form a key enzyme of the TCA pathway, *β*-hydroxyacyl-CoA, which enters the TCA cycle and participates in the conversion of glucose, fat, and proteins (Agrawal et al., 2018; Shimomura and Kitaura, 2018). The combined effect of multiple amino acid metabolism related genes enhances the level of energy metabolism for cell growth, promotes the cell growth of strain AKD4 at low temperatures, and maintains high nitrogen removal performance.

The TCA cycle is an important core metabolic pathway during microbial cell growth, providing substrates for cellular metabolic energy and biosynthetic reactions. Genes involved in the TCA cycle pathway directly related to cell growth showed a positive response under low-temperature stress. As shown Figure 3C, the transcriptome analysis of AKD4 revealed that the relative expression levels of the TCA cycle-related genes *gltA*, *fumC*, and *mdh* at 10°C were upregulated 2.46-, 1.25-, and 1.30-fold those of the same genes at 30°C, respectively. The *gltA* gene encoding citrate synthase was significantly upregulated at low temperatures, enhanced the TCA cycle, improved the efficiency of carbon dioxide conversion, and promoted the synthesis of L-glutamate to provide energy for cell growth (Figure 3D) (Buch et al., 2009). Metabolic analysis of *Propionibacterium freudenreichii* CIRM-BIA1^T^ showed that the relative expression level of the gene *fumC* was upregulated at 4°C, promoted the conversion of phosphoenolpyruvate, which is an additional substrate for gluconeogenesis, and provided metabolic energy for cell growth (Dalmasso et al., 2012). The upregulation of the relative expression levels of *fumC* and *mdh* genes of AKD4 at low temperatures can promote the activity of fumarase and phosphoenolpyruvate carboxylase, increase the supply of oxaloacetate, and then increase the production of succinate and amino acids, which can increase the metabolic flux of the carboxylation pathway and improve the efficiency of the TCA cycle for enhancing energy production and maintaining high cell activity in response to low-temperature stress.

#### 3.3.2 Expression of nitrogen metabolism-related genes

**Figure 4:**
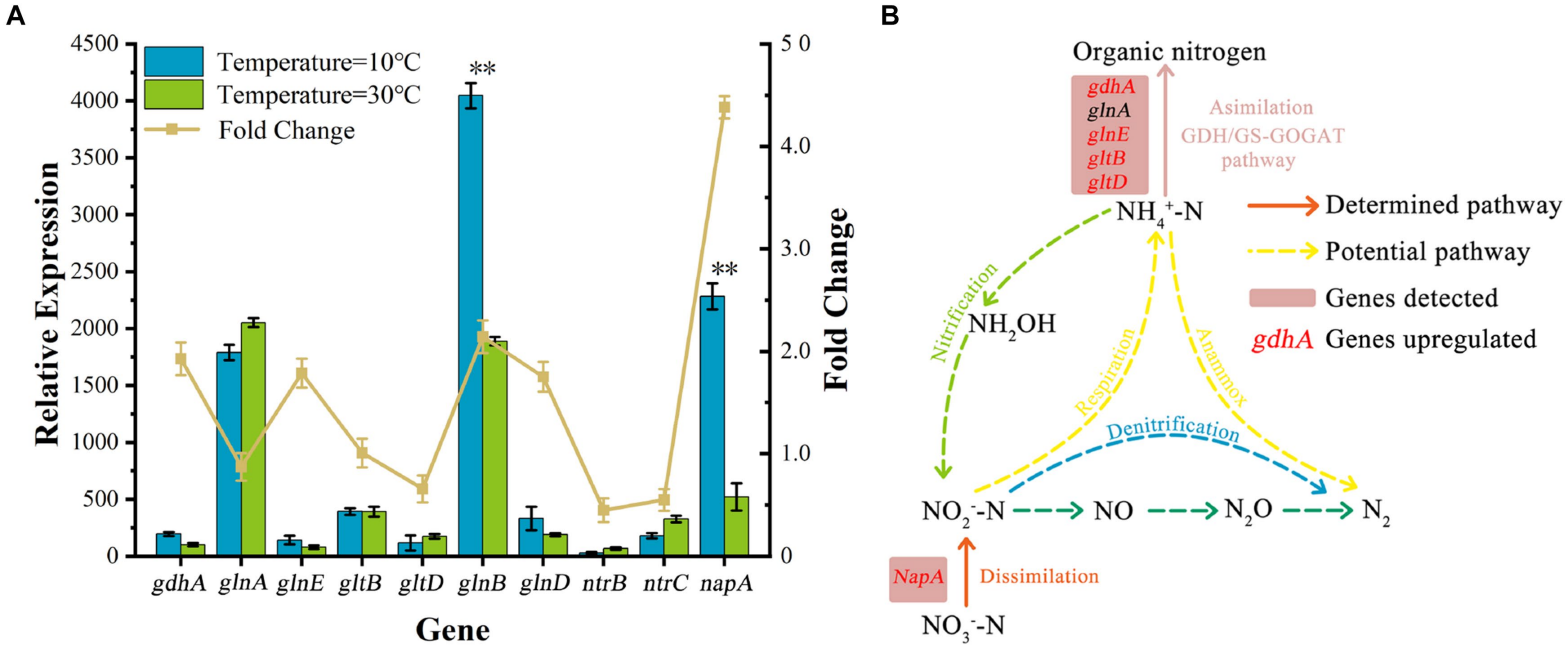
Expression analysis of nitrogen metabolism-related genes in cells grown at low temperatures. **(A)** The genes relevant to nitrogen metabolism in strain AKD4. **(B)** The typical gene of the nitrogen metabolism in strain AKD4.

Genomic functional annotation showed that the presence of nitrogen metabolism-related genes corresponding to nitrate reductase, glutamine synthetase, glutamate dehydrogenase, glutamate synthase, periplasmic nitrate reductase, and nitrogen-regulated proteins in AKD4. As shown in Figure 4B, other key pathway genes involved in nitrification (ammonia monooxygenase and hydroxylamine oxidase) and denitrification pathway genes were not detected in AKD4, consistent with the previous reports on the SND strains *K. pneumoniae* and *Klebsiella* sp. (Jin et al., 2019; Pal et al., 2015).

As to nitrogen metabolism, the two main pathways for assimilation of ammonium into organic compounds are glutamine synthetase (GS)-glutamate synthase (GOGAT) and glutamate dehydrogenase (GDH) pathway (Tyler, 1978). The GDH encoded by the *gdhA* gene can reversibly catalyze the generation of glutamate from NH_4_^+^–N and the catabolism of glutamate to NH_4_^+^–N at different nitrogen concentrations. The GOGAT encoded by *gltBD* can catalyze the reduction and synthesis of glutamine to glutamate and facilitates the entry of NH_4_^+^ into organic nitrogen pools. GS, encoded by *glnAE*, catalyzes the synthesis of glutamine from glutamine into glutamine and amino acids and provides energy for microbial growth. They form the GS-GOGAT cycle (Meng et al., 2016). The relative expression levels of *glnE*, *gltB* and *gdhA* genes were significantly upregulated at 10°C (1.75-, 1.01-, and 1.93-fold, respectively) compared with those at 30°C. Although the relative expression levels of *gl*nA and *gltD* decreased slightly, the relative expression levels of 87 and 66% were still maintained. This result demonstrated that GOGAT and GDH in AKD4 play important roles as ammonium assimilating enzymes, and their actions enhance microbial activity and ammonia assimilation capacity in low-temperature environments. In addition, the relative expression level of *gdhA* was higher than the expression levels of *glnAE* and *gltBD* at 10°C, indicating that AKD4 preferentially carried out ammonium assimilation through the GDH pathway. This result is consistent with the fact that SND strains preferentially assimilate ammonium via the GDH pathway at different ammonium concentrations (Jin et al., 2019). The genes encoded by *glnB* and *glnD* control the activity of the NtrB-NtrC two-component regulatory system (Dixon and Kahn, 2004). In this system, NtrB phosphorylates itself and then passes the phosphorylated group to NtrC, and the phosphorylated level of NtrC controls the expression levels of *glnA* and *ntrBC* manipulators. The expression levels of *glnB* and *glnD* genes were upregulated 2.14-and 1.75-fold, respectively, at 10°C compared with those at 30°C, indicating that the nitrogen-regulating system NtrC actively participates in regulating intracellular nitrogen source depletion at low temperatures. The expression levels of the genes encoding GS, GOGAT, and GDH were apparently upregulated at low temperatures, suggesting that these genes play important roles in ammonia assimilation in AKD4.

Nitrate dissimilation requires the participation of multiple enzymes to accomplish the first catalytic reduction of nitrate to nitrite. Three types of nitrate reductases, respiratory nitrate reductase, periplasmic nitrate isomerization reductase, and assimilatory nitrate reductase, were found in SND bacteria (Potter et al., 2001). Transcriptome results showed the presence of a periplasmic nitrate reductase (NAP) in AKD4. The NAP encoded by *napA* has a high affinity for nitrate and can synthesize and grow rapidly at low nitrate concentrations. As shown in Figure 4A, the relative expression of *napA* was upregulated 4.38-fold at 10°C compared with that at 30°C. The upregulation of *napA* expression at low temperatures suggests that it plays an essential role in nitrate isomerization and reduction. In SND bacteria, the NAP dominates under aerobic conditions and plays an essential part in the aerobic denitrification stage (Stewart et al., 2002). Periplasmic nitrate isomerization reductase, encoded by *napA*, converts nitrate into nitrite (Cui et al., 2021). The *napA* gene is subjected to a strong nitrate-inducing effect under low temperatures, and it can play a reducing role for nitrogen dissimilation. This may be the main reason for the low accumulation of NO_3_^−^–N in AKD4. Thus, the periplasmic nitrate reductase encoded by *napA* is involved in the major nitrate isomerization reduction.

## 4 Conclusion

In this study, a novel SND strain, AKD4, was isolated and screened from activated sludge, which could remove 97.85 and 92.45% of NH_4_^+^–N and TN at 10°C, respectively. The cell growth and nitrogen metabolism involved the pathway genes of AKD4 were elucidated by transcriptome analysis, which showed the remarkable upregulation of key genes related to the TCA pathway, ammonia assimilation, and dissimilation under low-temperature stress, preliminarily revealed the metabolic mechanism of this strain maintaining high growth activity and nitrogen removal efficiency at low-temperature. These observations should significantly enhance the understanding of the metabolic mechanisms at low-temperature-tolerant SND bacterium.

## Data Availability

The original contributions presented in the study are included in the article/supplementary material, further inquiries can be directed to the corresponding authors.

## Footnotes

^1^ http://bowtie-bio.sourceforge.net/index.shtml

^2^ https://blast.ncbi.nlm.nih.gov/Blast.cgi
